# *PTEN* Inhibits Cell Proliferation, Promotes Cell Apoptosis, and Induces Cell Cycle Arrest via Downregulating the PI3K/AKT/*hTERT* Pathway in Lung Adenocarcinoma A549 Cells

**DOI:** 10.1155/2016/2476842

**Published:** 2016-10-16

**Authors:** Xiao-Xiao Lu, Lan-Yu Cao, Xi Chen, Jian Xiao, Yong Zou, Qiong Chen

**Affiliations:** ^1^Department of Geriatrics, Xiangya Hospital of Central South University, Changsha, Hunan 410008, China; ^2^Department of Respiratory Medicine, Xiangya Hospital of Central South University, Changsha, Hunan 410008, China

## Abstract

*PTEN* plays an essential role in tumorigenesis and both its mutation and inactivation can influence proliferation, apoptosis, and cell cycle progression in tumor cells. However, the precise role of* PTEN* in lung cancer cells has not been well studied. To address this, we have generated lung adenocarcinoma A549 cells overexpressing wild-type or mutant* PTEN* as well as A549 cells expressing a siRNA directed toward endogenous* PTEN*. Overexpression of wild-type* PTEN* profoundly inhibited cell proliferation, promoted cell apoptosis, caused cell cycle arrest at G1, downregulated p-AKT, and decreased expression of the telomerase protein* hTERT*. In contrast, in cells expressing a* PTEN* directed siRNA, the opposite effects on cell proliferation, apoptosis, cell cycle arrest, p-AKT levels, and* hTERT* protein expression were observed. A549 cells transfected with a* PTEN* mutant lacking phosphatase activity (*PTEN*-C124A) or an empty vector (null) did not show any effect. Furthermore, using the PI3K/AKT pathway blocker LY294002, we confirmed that the PI3K/AKT pathway was involved in mediating these effects of* PTEN*. Taken together, we have demonstrated that* PTEN* downregulates the PI3K/AKT/*hTERT* pathway, thereby suppressing the growth of lung adenocarcinoma cells. Our study may provide evidence for a promising therapeutic target for the treatment of lung adenocarcinoma.

## 1. Introduction

Both oncogenes and tumor suppressors are potential targets in the field of tumor therapy. In organisms, the biological functions of oncogenes and antioncogenes mutually antagonize each other to regulate cell proliferation, differentiation, apoptosis, cell cycle, and angiogenesis. It has been found that dozens of genes are closely correlated with lung cancer, among which the oncogene* PTEN* (phosphatase and tensin homolog) and the tumor suppressor* hTERT* (human telomerase reverse transcriptase) have been extensively studied in the past few years [1–4].

The tumor suppressor gene* PTEN* encodes dual-specificity phosphatase that was first discovered in 1997 [5]. Inactivation of* PTEN* is a key event in tumorigenesis and tumor development, and in fact it has the highest frequency of mutation in cancer after the* P53* gene [6]. Currently, the tumor suppressing mechanism of the* PTEN* gene likely involves several candidate pathways, including the FAK pathway [7], the MAPK pathway [8, 9], and the PI3K/AKT pathway [10, 11]. Currently, the PI3K/AKT pathway is regarded as the key pathway by which* PTEN* exerts its antioncogenic effects.* PTEN* encodes a protein with lipid phosphatase activity, which can dephosphorylate PIP3 (phosphatidylinositol (3,4,5)-trisphosphate) to form PIP2 (phosphatidylinositol (4,5)-bisphosphate), thereby preventing growth factor signal transduction pathways regulated by PI3K/AKT. As a result,* PTEN* activity in tumor cells results in cell cycle arrest at the G1 phase and induction of apoptosis [10–12]. Moreover, the PI3K/AKT pathway plays an important central role in tumor progression, and it is closely associated with other pathways which control a wide variety of tumor related biological processes. Studies have found that both the FAK pathway and the MAPK pathway exert effects through the PI3K/AKT pathway and affect the activity of AKT [7–9]. In a study of ovarian cancer, it was found that the FAK pathway mediated the activation of multiple downstream substrates of AKT such as NF-*κ*B, promoted cell proliferation, and improved resistance to chemotherapeutic drugs [13].

Numerous studies have shown that telomerase activation is another extremely important step in the pathogenesis of lung cancer, and this depends on the activity of the* hTERT* gene [14, 15]. Despite its importance, the mechanisms of* hTERT* gene regulation have not been completely identified. However, it has been shown that there is a negative correlation between* hTERT* expression and* PTEN* expression in gastric cancer, liver cancer, and endometrial cancer [16].

*PTEN* has been found to be able to inhibit the activity of telomerase. The activity of telomerase declined significantly when wild-type* PTEN* gene segments were transfected into glioblastoma cells expressing a mutated form of* PTEN*. In addition, it was also shown in this study that the* PTEN*/PI3K/AKT pathway reduced the expression and function of* hTERT* [17]. A recent study also demonstrated that* PTEN* suppressed the phosphorylation of various tumor related proteins including hTERT through the PI3K/AKT pathway in renal cell carcinoma [18].

Our previous study has also found that the proliferative capacity of lung adenocarcinoma cells was significantly reduced when the exogenous wild-type* PTEN* gene was introduced into A549/CDDP cells, which are resistant to cisplatin. Simultaneously, G1 phase arrest was observed and the A549/CDDP cells displayed a considerable improvement in sensitivity to cisplatin [19]. In light of the above, it is reasonable for us to presume that the mechanism by which* PTEN* inhibits cell proliferation, promotes cell apoptosis, and induces cell cycle arrest in lung adenocarcinoma A549 cells may be related to the downregulation of* hTERT* expression and that the PI3K/AKT pathway might be implicated in this process.

## 2. Materials and Methods

### 2.1. Cell Line and Cell Culture

The human lung adenocarcinoma cell line (A549) was purchased from the Cell Center of Xiangya Medical College of Central South University (Changsha, Hunan, China). Cells were cultured in high-glucose Dulbecco's modified Eagle's medium (DMEM, Gibco, CA, USA) supplemented with 10% fetal bovine serum (Gibco, CA, USA), 100 U/mL penicillin, 100 *μ*g/mL streptomycin sulfate, and 2 mM glutamate in humidified atmosphere of 5% CO_2_ at 37°C.

### 2.2. Gene Transfection Technology

The null plasmid vector (pGFP), wild-type* PTEN* plasmid (pGFP-*PTEN*), and mutant-type* PTEN* plasmid (pGFP-*PTEN*-C124A) were generously supplied by Professor Kenneth M. Yamada, American National Institutes of Health. Plasmids were amplified by transformation in* E. coli*, extracted, and purified using a plasmid DNA extract kit (Macherey-Nagel, Germany). Insert sizes were confirmed by agarose gel electrophoresis.* PTEN* small interfering RNA (*PTEN*-siRNA) expressing green fluorescence protein (GFP) were synthesized by Cell Signaling Technology Co. Ltd. (Danvers, MA, USA). Each plasmid or siRNA was mixed with Lipofectamine 2000 reagent (Invitrogen, Carlsbad, CA, USA) following the manufacturer's instructions and was added to cells at 50%–70% confluence. After 4 h of incubation, the transfection medium was discarded, cells were washed three times with phosphate buffered saline (PBS), and the media were then replaced with serum-containing medium. After 48 h, the transfection efficiency and* PTEN* gene expression were estimated by fluorescence microscopy and Western blot analysis, respectively.

### 2.3. MTT

A549 cells were trypsinized and seeded into 96-well plates at a density of approximately 4000 cells per well. Twenty-four hours later, adherent cells were transfected with pGFP, pGFP-*PTEN*, pGFP-*PTEN*-C124A, and* PTEN*-siRNA and/or exposed to PI3K/AKT inhibitor (LY294002 10 *μ*M, Sigma-Aldrich, MO, USA), nontransfected A549 cells served as the control group. During the following one to six days, MTT reagent (3-[4,5-dimethylthiazol-2-yl]-2,5-diphenyltetrazolium bromide, Sigma-Aldrich, USA) was added to the cells (20 *μ*L (5 mg/mL) per well), and cells were then incubated for 4 h at 37°C. The cells media were removed and 150 *μ*L of DMSO was added to each well followed by gentle shaking of the plates to dissolve the formazan crystals. The optical density (OD) was then measured using a microplate reader at 490 nm.

### 2.4. Flow Cytometry Assay

The A549 cell apoptosis rate (expressed as a percentage) and cell cycle stage were measured using a PI (Propidium Iodide) staining kit (Sigma-Aldrich, MO, USA). Cells were collected and fixed in 70% ice cold ethanol overnight, stained with a mixture of PI and RNase in a 37°C water bath shielded from light for 30 min. Cells were then examined by fluorescence microscopy and the data were analyzed using WinMDI v 2.8 software.

### 2.5. Western Blot

A549 cells were trypsinized and washed three times with PBS before being lysed on ice for 30 min with RIPA lysis buffer containing protease inhibitors (Sigma-Aldrich, MO, USA). The lysate was centrifuged at 12,000 ×g at 4°C for 20 min. Protein concentrations were measured by the Bradford method. Total protein (50 *μ*g) for each sample was separated using 10% sodium dodecyl sulfate-polyacrylamide gel electrophoresis (SDS-PAGE) and transferred into polyvinylidene difluoride (PVDF) membranes (Merck Millipore Inc., MA, USA) and blocked for 1 h in TBST containing 5% skimmed milk. Proteins of interests were detected by incubation with the appropriate antibodies at 4°C overnight. The primary antibodies used were as follows: mouse anti-human PTEN (1 : 100 dilution), rabbit anti-human p-AKT (1 : 500 dilution), and rabbit anti-human hTERT (1 : 500 dilution) (Abcam Inc., Cambridge, MA, USA). Secondary antibodies (Santa Cruz Biotechnology, CA, USA) were incubated for 1 h at room temperature. Proteins were detected by ECL chemiluminescence. Image Lab 4.1 software was used to analyze the protein bands and relative protein expression level was normalized with reference to *β*-actin (Abcam Inc., Cambridge, MA, USA).

### 2.6. Real Time PCR

Total RNA was isolated from cells using Trizol reagent (Invitrogen, Carlsbad, CA, USA) according to the manufacturer's protocol. Total RNA (1 *μ*g) was reverse transcribed using a Reverse Transcription kit according to the manufacturers protocol (Promega, WI, USA) (reaction at 42°C for 40 min and denaturation at 99°C for 5 min). cDNA (2 *μ*L) was then amplified using the following conditions:* PTEN*: 30 cycles of denaturation for 50 s at 94°C; annealing for 45 s at 60°C; and extension for 7 min at 72°C and* hTERT*: 30 cycles of denaturation for 30 s at 94°C; annealing for 30 s at 60°C; and extension for 45 s at 72°C. The PCR product for each sample was separated by 1.5% agarose gel electrophoresis at 80 V. The *β*-actin or GAPDH mRNA expression level was employed as an internal control. The sequence of the primer pairs used is listed below:

 
*PTEN*: 5′-GTAAGGACCAGAGACAAAAAG-3′ and 3′-CTTTTTTAGCATCTTGTTCTG-5′
 
*hTERT*: 5′-TTTCTGGAGCTGCTTGGGAA-3′ and 3′-GAAGAGCCTGAGCAGCTCGA-5′
 
*β*-Actin: 5′-TGAAGTGTGACGTGGACATC-3′ and 3′-GGAGGAGCAATGATCTTGAT-5′
 GAPDH: 5′-ACCACAGTCCATGCCATCAC-3′ and 3′-TCCACCACCCTGTTGCTGTA-5′

### 2.7. Statistical Analysis

GraphPad Prism 5 software was used for statistical analysis. All data were obtained from at least three independent experiments. Data in each group is presented as the mean ± standard deviation (*χ* ± SD), and the difference between groups was analyzed by analysis of variance (ANOVA) or a two-tailed Student's *t*-test. *p* value less than 0.05 was counted as being statistically different.

## 3. Results

### 3.1. Effects of Different* PTEN* Phenotypes on A549 Cell Proliferation, Apoptosis, and Cell Cycle Progression

**Figure 1:**
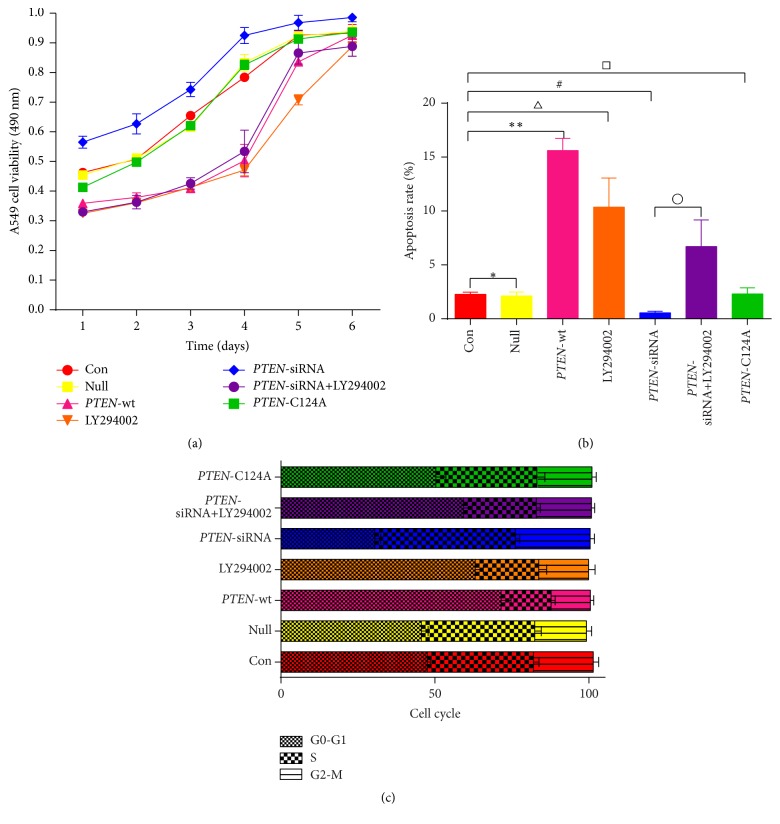
Effects of different* PTEN* phenotypes on A549 cell proliferation, apoptosis, and cell cycle progression. A549 cells were transfected with pGFP (null), pGFP-*PTEN* (*PTEN-*wt), pGFP-phosphatase-dead* PTEN* (*PTEN*-C142A), or* PTEN*-siRNA in the presence or absence of LY294002 (10 *μ*M) (*PTEN*-siRNA and* PTEN*-siRNA+LY294002). Untransfected A549 cells were used as the control group (con) and these cells were also treated with LY294002 (10 *μ*M) (LY294002). Cell viability (a), the cellular apoptosis rate (b), and cell cycle progression (c) were measured by MTT and flow cytometry, respectively (^*∗*^ *p* > 0.05, ^*∗∗*^ *p* < 0.05, ^△^ *p* < 0.05, ^#^ *p* < 0.05, ^□^ *p* > 0.05, and ^∘^ *p* < 0.05).

Our previous report demonstrated that* PTEN* could regulate cell proliferation, cell cycle, and drug sensitivity to cisplatin in A549/CDDP cells. It has been documented that A549 cells express wild-type* PTEN* [20, 21]. To further investigate whether different* PTEN* phenotypes have the same antitumor effect on A549 cells, vector alone (null), wild-type* PTEN*, a phosphatase-dead* PTEN* mutant (*PTEN*-C124A), and* PTEN*-siRNA were transfected separately into A549 cells using untransfected A549 cells as the control group. Cell proliferation was assessed using an MTT assay whereas cell apoptosis and cell cycle progression were assessed using a flow cytometry assay. As shown in Figure 1(a), the cell growth curves showed logarithmic growth from the first day to the fourth day; after this time, cell proliferation began to plateau. Over the first four days of growth, it was readily apparent that cell proliferation was suppressed in cells overexpressing wild-type* PTEN* compared to control cells. In contrast, cell proliferation in the* PTEN*-siRNA cells was significantly enhanced (*p* < 0.05). Furthermore, there was no apparent change in cell proliferation in both the null cells and the phosphatase-dead* PTEN* cells compared with the control cells (*p* > 0.05). The PI3K/AKT pathway can be blocked by using the AKT inhibitor LY294002. Our results showed that, in control cells, LY294002 treatment also suppressed cell proliferation. In contrast, the enhancement of proliferation seen in the* PTEN*-siRNA treated cells was blocked by LY294002 treatment.

The flow cytometry data demonstrated that the rate of cell apoptosis was significantly enhanced in cells overexpressing wild-type* PTEN*, compared with the control cells (15.6 ± 1.14% versus 2.27 ± 0.21%, ^*∗∗*^ *p* < 0.05). LY294002 of control cells also increased the level of apoptosis to levels similar to those seen in wild-type* PTEN* overexpressing cells (10.36 ± 2.7% versus 2.27 ± 0.21%, ^△^ *p* < 0.05). Similarly, compared with the control cells, there was an increased proportion of G0-G1 phase cells in the wild-type* PTEN* group (71.5 ± 3.22% versus 47.5 ± 1.03%, ^*∗∗*^ *p* < 0.05) and LY294002 treatment of control cells also similarly increased the proportion of cells in the G0-G1 phase (63.1 ± 2.64% versus 47.5 ± 1.03%, ^△^ *p* < 0.05). On the contrary, both the rate of cell apoptosis (0.55 ± 0.15% versus 2.27 ± 0.21%, ^#^ *p* < 0.05) and the proportion of cells in the G0-G1 phase (30.5 ± 1.89% versus 47.5 ± 1.03%, ^#^ *p* < 0.05) were significantly reduced in the* PTEN*-siRNA group compared to the control group. Treatment of* PTEN*-siRNA cells with LY294002 attenuated both the decrease in rate of cell apoptosis (6.71 ± 2.47 versus 0.55 ± 0.15%, ^∘^ *p* < 0.05) and the decrease in percent of cells in the G0-G1 phase (59.3 ± 2.77% versus 30.5 ± 1.89%, °*p* < 0.05) caused by the* PTEN*-siRNA. Moreover, compared with control group, there was no statistical difference in the rate of cell apoptosis for the null cells (2.13 ± 0.36% versus 2.27 ± 0.21%, ^*∗*^ *p* > 0.05) or the phosphatase-dead* PTEN* cells (2.31 ± 0.57% versus 2.27 ± 0.21%, ^□^ *p* > 0.05). Similar observations were made for the percentage of cells in the G0-G1 phase (45.6 ± 2.22% (null) versus 47.5 ± 1.03% (control), ^*∗*^ *p* > 0.05, and 50.3 ± 3.27% (phosphatase-dead* PTEN*) versus 47.5 ± 1.03% (control), ^□^ *p* > 0.05) (Figures 1(b) and 1(c)). These results suggested that wild-type* PTEN*, but not a phosphatase-dead* PTEN* mutant, was capable of suppressing cell proliferation, inducing cell apoptosis, and arresting the cell cycle in adenocarcinoma A549 cells and that the PI3K/AKT pathway possibly participated in these effects.

### 3.2. *PTEN* Negatively Regulates* hTERT* by Inhibiting the PI3K/AKT Pathway

As shown in Figure 1, the role of* PTEN* as a tumor suppressor in A549 cells has been convincingly demonstrated by its effects on cell proliferation, apoptosis, and cell cycle arrest. In light of previous reports showing that* PTEN* can negatively regulate* hTERT* in hepatocellular carcinoma, renal carcinoma, and glioma cells and that PI3K/AKT acts downstream of* PTEN*, we hypothesized that* PTEN* might inhibit tumor progression by suppressing the PI3K/AKT/*hTERT* pathway.

**Figure 2:**
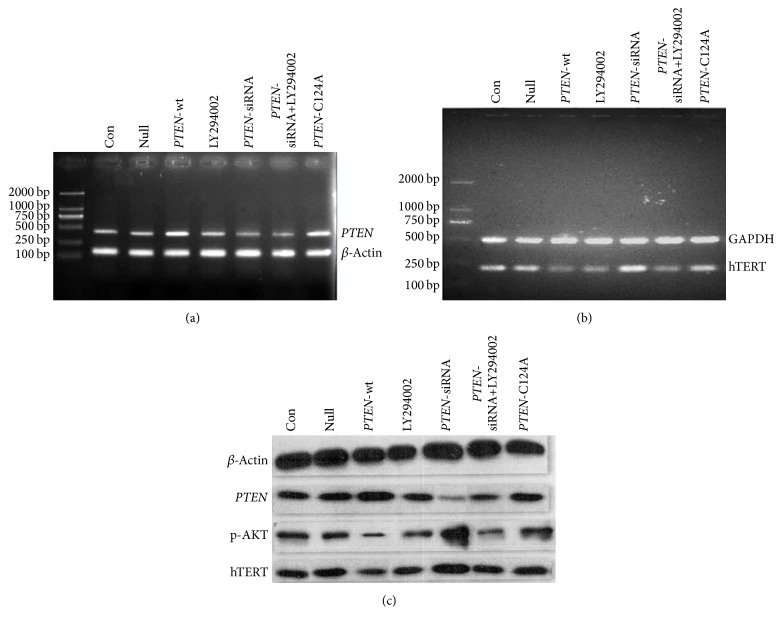
*PTEN* negatively regulates* hTERT* by inhibiting the PI3K/AKT pathway. Untransfected A549 cells (con) or A549 cells transfected with either pGFP (null), pGFP-*PTEN *(wild-type* PTEN*), pGFP-*PTEN*-C124A (phosphatase-dead* PTEN*), or* PTEN*-siRNA and/or exposed to the AKT inhibitor (LY294002 10 *μ*M) were examined for the expression of* PTEN* (a) and* hTERT* (b) mRNA by RT-PCR, as well as for the protein levels of* PTEN*, hTERT, and p-AKT (c) by Western blot.

In order to investigate this hypothesis, we examined the mRNA and protein expression level of* PTEN*,* hTERT*, and p-AKT in cells expressing different levels of* PTEN*, or a phosphatase-dead* PTEN* mutant, along with the combination of treatment with PI3K/AKT pathway inhibitor LY294002. As shown in Figures 2(a) and 2(c), there was no detectable difference in the mRNA and protein expression level of* PTEN* between the control group and the null plasmid transfected cells, which demonstrated that the plasmid vector did not affect* PTEN* expression. As expected, upregulation of* PTEN* at both the mRNA and protein levels could be observed in cells transfected with wild-type* PTEN* as well as in cells transfected with the phosphatase-dead mutant* PTEN*. In cells transfected with a* PTEN*-siRNA, also as expected, lower levels of* PTEN* could be detected at both the mRNA and protein levels. Treatment of cells with LY294002 did not affect* PTEN* expression, which we believe is evidence that PI3K/AKT is located downstream of* PTEN*.

In this study, we also found that the mRNA and protein levels of* hTERT* were considerably lower in A549 cells transfected with wild-type* PTEN* than in the control group (*p* < 0.05). Conversely, dramatically elevated* hTERT* mRNA and protein level could be observed in A549 cells transfected with a* PTEN*-siRNA compared to the control group (*p* < 0.05). These results confirm that* PTEN* also negatively regulates* hTERT* in lung adenocarcinoma cells. Although the* PTEN* mRNA and protein levels increased after A549 cells were transfected with the phosphatase-dead* PTEN* mutant (*PTEN-C124A*), this was not accompanied by a change in* hTERT* expression levels (*p* > 0.05), confirming that the phosphatase-dead* PTEN* is nonfunctional and that only wild-type* PTEN* plays a role in suppressing* hTERT* expression (see Figures 2(b) and 2(c)).

Intriguingly, we also found that, compared to control cells, the phosphorylation levels of AKT were decreased in cells overexpressing wild-type* PTEN*, while they were increased in cells transfected with a* PTEN*-siRNA. These data highlight the fact that PI3K/AKT is inhibited by wild-type* PTEN* but not by a phosphatase-dead mutant* PTEN* in these lung adenocarcinoma cells. Furthermore, we demonstrated that the PI3K/AKT pathway blocker LY294002 could mimic the effects of wild-type* PTEN* overexpression. Both the mRNA and the protein level of* hTERT* were remarkably lower in A549 cells after exposure to LY294002 alone. Treatment of cells expressing a* PTEN* directed siRNA with LY294002 abrogated the upregulation of* hTERT* expression caused by the* PTEN*-siRNA (*p* < 0.05) (see Figures 2(b) and 2(c)). Collectively, these data, as well as previously published results, reveal that the* PTEN *gene act as a tumor suppressor by inhibiting cell proliferation, promoting cell apoptosis, and inducing cell cycle arrest in human lung adenocarcinoma* in vitro* and that these effects were somewhat mediated through a negative regulation of the PI3K/AKT/*hTERT* pathway.

## 4. Discussion

Although it is well known that the* PTEN* gene plays a pivotal role in suppressing tumor development by negatively regulating the* hTERT* gene in multiple tumor cells, it remains to be discovered whether similar effects of* PTEN* on the* hTERT* gene also occur in lung cancer. In this study, we focused on the human lung cancer A549 cell line and evaluated the function of* PTEN* gene on cell proliferation, apoptosis, and cell cycle arrest by over- or underexpressing wild-type* PTEN* and comparing effects to those seen with phosphatase-dead mutant* PTEN*.

Data from previous studies in glioma, endometrial cancer, and other tumors suggested that the exogenous wild-type* PTEN* gene can profoundly inhibit the growth of tumor cells, promote cellular apoptosis, and cause cell cycle arrest at the G1 phase [22–27]. Our study, in lung cancer cells, now also confirms these findings. Although the A549 cell line expresses low levels of the* PTEN* gene [28, 29], these levels can be further increased by transfection with the exogenous wild-type* PTEN* and this leads to suppression of cell proliferation. Bruni and his colleagues [30] found that the expression of exogenous wild-type* PTEN* can inhibit tumor growth independent of whether the cells express the endogenous* PTEN* gene or not and that the inhibitory effect is more obvious when the endogenous* PTEN* gene is completely deleted. On the contrary, cell proliferation and apoptosis are unchanged in A549 cells expressing a phosphatase-dead mutant* PTEN* gene (*PTEN*-C124A) [5, 31, 32]. These data highlight the fact that the phosphatase activity of the* PTEN* gene is indispensable for the effects of* PTEN* on restraining cancer cell growth as well as promoting apoptosis.

Here, we further analyzed the relationship between the* PTEN* and* hTERT* genes in A549 cells. The results showed that the mRNA and protein levels of* PTEN* increased after transfection of lung adenocarcinoma A549 cells with the wild-type* PTEN* plasmid and that at the same time* hTERT* mRNA and protein expression levels were reduced. However, there were no obvious changes of the* hTERT* mRNA and protein expression observed in A549 cells transfected the mutant-type* PTEN* plasmid. In addition, we found that the* hTERT* mRNA and protein expression levels increased when the* PTEN* gene was silenced using a* PTEN* directed siRNA. These data suggest that the expression level of* hTERT* is inversely associated with the activity of the wild-type* PTEN* gene.

The* hTERT* gene is considered to be the key rate limiting factor, which regulates the activity of telomerase, and its expression level may indirectly reflect the activity of telomerase. It plays a critical role in the process of development of tumor by inducing the clonal growth of cell by bypassing the process of replicative senescence thereby contributing to malignant immortalization [2, 14, 18]. An earlier study has reported that, in approximately 85% of people with cancer, telomerase activity could be detected in tumor tissues, whereas telomerase activity was detected in only about 4% of normal tissues adjacent to the tumor or in benign lesions [33]. Increased telomerase activity can suppress tumor cell apoptosis by affecting DNA stability and through signal transduction pathways [34]. Concordantly, it has also been demonstrated that the reduction of* hTERT* expression using an* hTERT* siRNA inhibited telomerase activity and accelerated cell apoptosis in lung cancer [35], further strengthening our hypothesis that* PTEN* suppresses the activity of telomerase by decreasing the expression of* hTERT*, leading to the inhibition of cell proliferation and the promotion cell apoptosis in lung adenocarcinoma A549 cells.

The* PTEN* gene participates in a myriad of physiological and pathological processes and its biological effects mainly depend on its interaction with downstream signaling molecules. In view of the evidence that the* PTEN*-PI3K/AKT-*hTERT* axis is a commonly operative pathway in various carcinoma models, we presumed that the PI3K/AKT pathway might be implicated in the regulation of* hTERT* by* PTEN* in lung cancer. Our results show that the levels of phosphorylated AKT (p-AKT) were reduced and that cell proliferation was inhibited in A549 cells overexpressing wild-type* PTEN* compared to control cells. The opposite effects were observed in cells transfected with a* PTEN* directed siRNA. In order to clarify if AKT plays a central role in this process, we used LY294002 a PI3K/AKT pathway inhibitor that inhibits the AKT pathway [36]. As expected, following LY294002 treatment, the AKT protein phosphorylation levels decreased. Importantly, LY294002 treatment of A549 cells decreased cellular proliferation, increased the apoptosis rate, and increased the percentage of cells arrested at G1. These effects were very similar in magnitude to those observed by overexpression of* PTEN* itself. LY294002 treatment of cells expressing a* PTEN* directed siRNA also profoundly suppressed the enhanced cell proliferation, reduced cellular apoptosis, and decreased cell cycle arrest seen in the* PTEN*-siRNA transfected cells alone. Based on these data, we have verified that the PI3K/AKT pathway indeed plays a central role in the mediating of the effects of PTEN on cell proliferation, apoptosis, and cell cycle arrest in A549 cells.

As an essential signal transduction protein, AKT plays a central role in the PI3K/AKT cell survival signaling pathway in cancer cells, and it also plays a crucial role in the cell survival mechanisms and signal transduction pathways mediating tumorigenesis. Only the phosphorylated form of AKT (namely, p-AKT) has biological activity [37]. This can be attributed to two factors. First, phosphorylated AKT prevents cell apoptosis by several mechanisms, including direct phosphorylation, the preapoptosis protein BAD, direct phosphorylation of the forkhead transcription factor FKHR-L1, and reducing the protease activity of caspase-9. AKT activation depends on the production of PIP3 by PI3K, and* PTEN* dephosphorylates PIP3 at the 3′ position, thereby preventing phosphorylation and activation of AKT and subsequently leading to the activation of downstream apoptosis signaling pathways and an increase in apoptosis [38–41]. Second,* PTEN* is also able to downregulate the cyclin-dependent kinase (CDK) by virtue of its protein phosphatase activity. In addition,* PTEN* can block the phosphorylation of CDKIs (cyclin-dependent kinase inhibitors) by AKT, allowing the entry of CDKIs into the nucleus and thereby suppressing the function of CDKs and impeding cell cycle progression [34, 42–44].

In addition, data from our study showed that* hTERT* mRNA and protein expression were clearly reduced when we utilized LY294002 to block the PI3K/AKT pathway in A549 cells. This same reduction was also seen in A549 cells overexpressing wild-type* PTEN*. Treatment of A549 cells expressing a* PTEN* directed siRNA with LY294002 abrogated the upregulation of* hTERT* expression seen in untreated cells. These data, along with the data on cell proliferation, apoptosis, and cell cycle arrest, show that inhibition of the PI3K/AKT pathway by LY294002 can mimic the anticancer effect of wild-type* PTEN*. In agreement with the present study, another study using the cervical cancer HeLa cell line treated with LY294002 showed that expression of* hTERT* can also be affected by inhibition of the PI3K/AKT pathway. Another study in endometrial carcinoma also showed that* PTEN* could reduce* hTERT* mRNA expression by the PI3K/AKT pathway [45, 46]. Past studies have pointed out that* hTERT*, as a telomerase complex catalytic unit, can be activated in a variety of ways, including PKB/AKT phosphorylation [10]. Although the mechanism by which the PI3K/AKT pathway upregulates hTERT is unclear, it has been suggested that it could be mediated by direct phosphorylation of serine residues in hTERT by p-AKT [13].

## 5. Conclusions

In light of the above findings, we conclude that downregulation of the PI3K/AKT/*hTERT* pathway may be one of the mechanisms by which the* PTEN* gene can act as a tumor suppressor in lung adenocarcinoma A549 cells. Further studies in animal model will still be needed to pave the way for future lung cancer treatment and prevention strategies.
